# Breast Cancer Patients With Positive Apical or Infraclavicular/Ipsilateral Supraclavicular Lymph Nodes Should Be Excluded in the Application of the Lymph Node Ratio System

**DOI:** 10.3389/fcell.2022.784920

**Published:** 2022-04-04

**Authors:** Zhe Wang, Wei Chong, Huikun Zhang, Xiaoli Liu, Yawen Zhao, Zhifang Guo, Li Fu, Yongjie Ma, Feng Gu

**Affiliations:** ^1^ Department of Tumor Cell Biology, Tianjin Medical University Cancer Institute and Hospital, National Clinical Research Center for Cancer, Tianjin, China; ^2^ Tianjin’s Clinical Research Center for Cancer, Tianjin Medical University Cancer Institute and Hospital, Tianjin, China; ^3^ Key Laboratory of Cancer Prevention and Therapy, Tianjin, China; ^4^ Key Laboratory of Breast Cancer Prevention and Therapy, Ministry of Education, Tianjin Medical University, Tianjin, China; ^5^ Department of Breast Cancer Pathology and Research Laboratory, Tianjin Medical University Cancer Institute and Hospital, Tianjin, China

**Keywords:** breast cancer, cut-off values, lymph node ratio, prognosis, pN stage

## Abstract

**Aim:** Increasing studies have demonstrated lymph node ratio (LNR) to be an accurate prognostic indicator in breast cancer and an alternative to pN staging; however, the AJCC-TNM staging system classified apical or infraclavicular/ipsilateral supraclavicular lymph node-positive (APN(+)) patients with a worse prognosis as the pN3 stage. Until now, different reports on LNR in breast cancer have ignored this possibility. Consequently, it is necessary to discuss the role of APN(+) patients in the LNR system to obtain a precise LNR that predicts the prognosis accurately.

**Materials and Methods:** We collected data on 10,120 breast cancer patients, including 3,936 lymph node-positive patients (3,283 APN(−) and 653 APN(+) patients), who visited our hospital from 2007 to 2012. Then we applied X-tile analysis to calculate cut-off values and conduct survival analysis and multivariate analysis to evaluate patients’ prognosis.

**Results:** We confirmed that some APN(+) patients were mis-subgrouped according to previously reported LNR, indicating that APN(+) patients should be excluded in the application of LNR to predict prognosis. Then we applied X-tile analysis to calculate two cut-off values (0.15 and 0.34) for LNR-APN(−) patients and conducted survival analysis and found that LNR-APN(−) staging was superior to pN staging in predicting the prognosis of APN(−) breast cancer patients.

**Conclusion:** From this study, we conclude that excluding APN(+) patients is the most necessary condition for effective implementation of the LNR system. LNR-APN(−) staging could be a more comprehensive approach in predicting prognosis and guiding clinicians to provide accurate and appropriate treatment.

## Introduction

The latest American Joint Committee on Cancer (AJCC) staging system recommends that pathologists evaluate the prognosis of patients by pN stage (Greene, 2002; Singletary et al., 2002). However, this classification only considered the number of positive lymph nodes and did not take the total number of lymph nodes into account. In recent years, emerging researchers have proposed lymph node ratio (LNR), the number of involved positive lymph nodes divided by the total number of lymph nodes examined, to be a better prognostic indicator than absolute lymph node number (Woodward et al., 2006; Vinh-Hung et al., 2009; Yang et al., 2017).

Remarkably, the WHO classification of breast tumors and AJCC demonstrated that patients with apical or infraclavicular/ipsilateral supraclavicular lymph node metastasis should be classified into the pN3 stage according to the traditional pN staging system, regardless of the status of lower-level metastatic lymph nodes, which were considered to exhibit a poor prognosis (Güven et al., 2007; Mary et al., 2009; Shalaka et al., 2019). Until now, reports on LNR in breast cancer from different research groups did not focus on the impact of APN(+) on the LNR system (Vinh-Hung et al., 2009; Kim et al., 2011; Duraker et al., 2013; Wu et al., 2013).

In order to illustrate the role of APN(+) in the LNR system and obtain the most precise LNR, we collected data on 10,120 patients diagnosed with breast cancer from 2007 to 2012 in our hospital (Tianjin Medical University Cancer Institute and Hospital). A total of 3,936 patients had positive lymph nodes, including 3,283 APN(−) and 653 APN(+) patients. We found that APN(+) patients had a significantly worse prognosis than APN(−) breast cancer patients in the same group according to previously reported LNR, indicating that APN(+) patients should be excluded in the application of the LNR system to predict prognosis. Then, we applied X-tile analysis to the data on the cohort of APN(−) patients to calculate two cut-off values (0.15 and 0.34) based on overall survival of these patients and defined the group as LNR-APN(−). Survival analysis further revealed that LNR-APN(−) staging was superior to pN staging in predicting the prognosis of APN(−) breast cancer patients.

## Materials and Methods

### Ethical Statement and Patient Selection

A total of 10,120 patients were diagnosed with breast cancer, including 6,184 patients with negative axillary lymph nodes and 3,936 patients with positive axillary lymph nodes, from January 2007 to December 2012 according to data from the archives of the Department of Breast Cancer Pathology and Research Laboratory, Tianjin Medical University Cancer Institute and Hospital. Patients with positive lymph nodes were further classified into APN(−) (3,283 patients) and APN(+) (653 patients). This study was approved by the Institutional Ethics Committee of Tianjin Medical University Cancer Institute and Hospital (bc2017019), and each participant signed an informed consent document.

All patients who underwent axillary lymph node dissection and received radical mastectomy or modified radical mastectomy were selected. Surgical specimens were then prepared for histological analysis: the specimens were fixed in 10% formaldehyde, and 2-µm sections were taken every 1.5 mm. Two experienced pathologists evaluated the status of the lymph nodes based on the World Health Organization histological classification of breast tumors. Metastatic nests >0.2 mm in diameter were scored as lymph node-positive metastases. After surgery, all patients were administered adjuvant chemotherapy and/or radiotherapy and/or endocrine therapy according to the National Comprehensive Cancer Network (NCCN) guidelines. Patients with multisource tumor, bilateral breast cancer, and loss to follow-up were excluded. We defined loss of follow-up as patients lost to follow up after being discharged from the hospital. Lumpectomy is the common treatment for early-stage breast cancer; most of these patients who underwent sentinel lymph node biopsy (SLNB) usually have a small number of lymph nodes. So, patients who received lumpectomy were excluded from the study. Information recorded for each patient included age at diagnosis, year of surgery, histologic features of the tumor, lymph node status, and survival. The median follow-up period was 81 (range 1–149) months.

### Cut-Off Values of LNR-APN(−) Staging

Positive lymph nodes identified on histopathological examination were classified according to the eighth edition of the AJCC staging system into three stages: pN1 (one to three positive lymph nodes), pN2 (four to nine positive lymph nodes), and pN3 (more than nine positive lymph nodes and at least one positive apical or infraclavicular/ipsilateral supraclavicular lymph node). LNR was calculated by the number of positive lymph nodes/total lymph nodes examined in node-positive patients. We excluded APN(+) breast cancer patients and obtained the optimal cut-off values of LNR-APN(−) staging by using the X-tile plots (X-tile software 3.6.1, Yale University, New Haven, CT, United States) in terms of overall survival. X-tile is a bioinformatics tool for biomarker assessment and outcome-based cut-point optimization (Robert et al., 2004). The X-tile plot shows the robustness of the relationship between LNR-APN(−) and patient outcome *via* construction of a two-dimensional projection of every possible subpopulation. Chi-square values were calculated for every possible division of LNR-APN(−), and the program selected the optimal division of LNR-APN (−) by choosing the highest chi-square value. The interval between the given set of divisions was 0.01. Therefore, the X-tile program divided the entire cohort into three subgroups based on the ratio of positive lymph nodes, which were LNR1-APN(−) (<0.15), LNR2-APN(−) (0.15–0.34), and LNR3-APN(−) (>0.34).

### SEER Database

We collected information on female breast cancer patients diagnosed between 1 January 2010 and 31 December 2012 from the SEER (Surveillance, Epidemiology, and End Results) database. Patients diagnosed with breast cancer before 2010 were excluded from this study because of unavailability of HER2 data. A total of 10,163 patients who met the following criteria were included: breast cancer as the primary cancer, unilateral breast cancer, received radical mastectomy or modified radical mastectomy, one or more involved lymph nodes, one or more positive lymph nodes, and known tumor size.

### Statistical Analysis

Overall survival (OS) and disease-free survival (DFS) were the main endpoints of this trial. The follow-up interval for OS and DFS was calculated in months. OS was defined as the time between the date of diagnosis and the date of death from any cause or the date of last follow-up. DFS was defined as the time from the date of diagnosis to the date of the first locoregional recurrence or/and distant metastasis, or the last follow-up date. OS and DFS curves were estimated using the Kaplan–Meier method and compared by the log-rank test, and the chi-square test was used to compare differences between groups. The independent prognostic effect of LNR-APN(−) was investigated using the Cox regression analysis, adjusting for age at diagnosis, histological grade, pT stage, and pN stage. Hazard ratios (HRs) along with 95% confidence intervals (95% CIs) were calculated. Two-tailed *p* values of less than 0.05 were considered to be statistically significant. All statistical analyses were performed using the SPSS version 26.0 software package for Windows (IBM SPSS Statistics, Chicago, IL, United States).

## Results

### Patients and Characteristics

**FIGURE 1:**
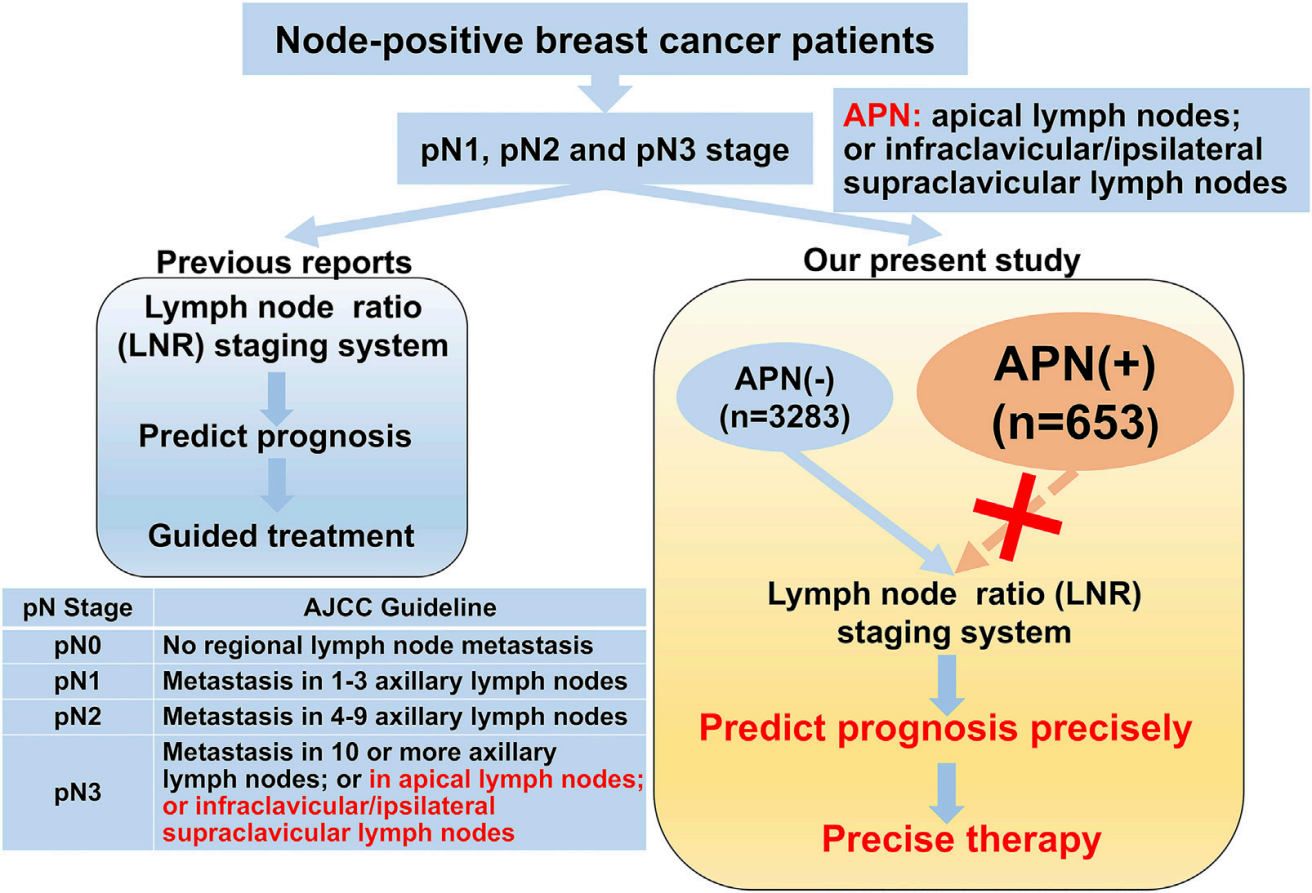
Graphical abstract of the lymph node ratio system.

The graphical abstract is shown in Figure 1. A total of 10,120 patients were diagnosed with breast cancer from 2007 to 2012 in Tianjin Medical University Cancer Institute and Hospital, and the clinicopathologic characteristics of the breast cancer patients are summarized in Table 1. Of the 10,120 breast cancer patients, 6,184 (61.1%) and 3,936 (38.9%) patients were node-negative and node-positive, respectively. The mean number of dissected lymph nodes was 23.1. Based on the eighth edition of the AJCC staging system, 2,213 patients were classified as pN1 (21.9%), 804 patients as pN2 (7.9%), and 919 patients as pN3 (9.1%). The median follow-up time for all 10,120 patients was 81 (range 1–149) months. We also present the detailed description of abbreviations in Supplementary Table S1.

**TABLE 1:** Clinicopathologic characteristics of breast cancer patients in the TCIH database (*n* = 10,120).

Characteristic	Number of patients (*n* = 10,120)	%
Age (years)		
<50	4,636	45.8
≥50	5,484	54.2
Histopathologic type		
Invasive ductal	7,500	74.1
Invasive micropapillary	335	3.3
Invasive lobular	249	2.5
Mucinous	136	1.3
Other types	1,900	18.8
Histological grade		
I	985	9.7
II	6,150	60.8
III	1,221	12.1
Unknown	1,764	17.4
Estrogen receptor^a^		
Negative	3,034	34.3
Positive	5,802	65.7
Progesterone receptor^a^		
Negative	3,525	39.9
Positive	5,302	60.1
HER2 expression^a^		
0 and 1+	6,282	71.3
2+	1,733	19.7
3+	790	9.0
pT stage		
pT1	4,905	48.5
pT2	4,753	47.0
pT3	386	3.8
pT4	76	0.7
Number of lymph nodes removed		
1–3	42	0.4
4–9	223	2.2
≥10	9,855	97.4
pN stage		
pN0	6,184	61.1
pN1	2,213	21.9
pN2	804	7.9
pN3	919	9.1

TCIH, Tianjin Medical University Cancer Institute and Hospital.

a Some data missing.

### Some APN(+) Patients With Poor Prognosis Were Mis-Subgrouped Into Low LNR Stage Using the LNR System

**FIGURE 2:**
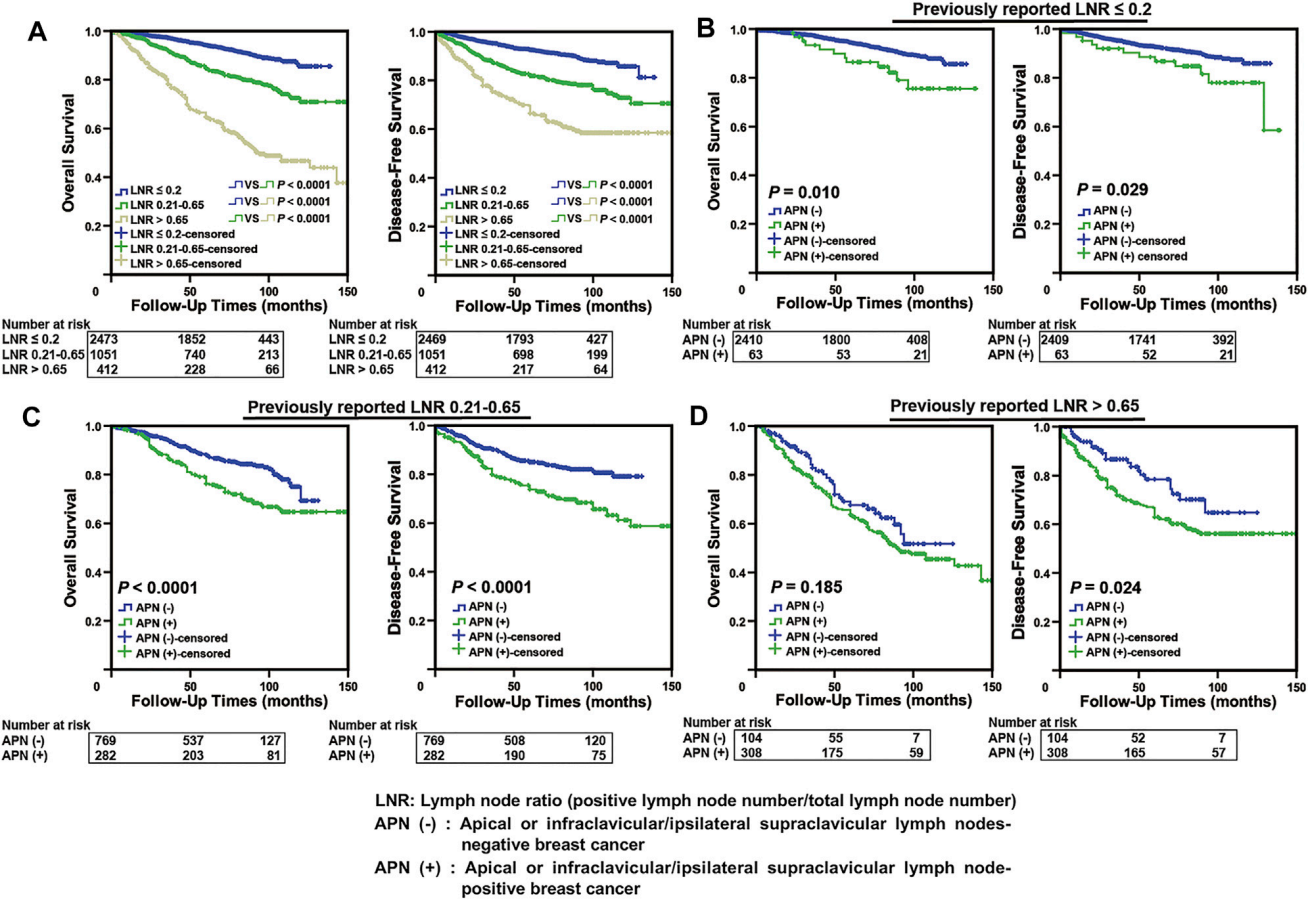
Some APN(+) patients with poor prognosis were mis-subgrouped in the low LNR stage using the LNR system. **(A)** Kaplan–Meier analysis in our breast cancer cohort according to previously reported LNR (*n* = 3,936). **(B)** Comparison of Kaplan–Meier curves of APN(−) and APN(+) breast cancer patients based on previously reported LNR ≤ 0.2 (*n* = 2,473, OS: *p* = 0.010, DFS: *p* = 0.029). **(C)** Comparison of Kaplan–Meier curves of APN(−) and APN(+) breast cancer patients based on previously reported LNR 0.21–0.65 (*n* = 1,051, OS: *p* < 0.0001, DFS: *p* < 0.0001). **(D)** Comparison of Kaplan–Meier curves of APN(−) and APN(+) breast cancer patients based on previously reported LNR > 0.65 (*n* = 412, DFS: *p* = 0.024).

We applied the Kaplan–Meier survival analysis to our cohort based on the representative previously reported LNR (cut-off values: 0.2 and 0.65) (Vinh-Hung et al., 2009; Wu et al., 2013; Wu et al., 2015; Quintyne et al., 2017) and found that there was a significant difference in survival among different groups (*p* < 0.0001, Figure 2A). In the subgroup analysis, APN(+) patients were found to have a significantly worse prognosis than APN(−) patients in the LNR1 (LNR ≤ 0.2) and LNR2 (LNR 0.21–0.65) groups (*p* < 0.05, Figures 2B,C). In the LNR3 (LNR > 0.65) group, there was no difference in OS between APN(+) and APN(−) breast cancer patients, but a significant difference was noted in DFS, considering the poor prognosis within this group (Figure 2D). These results indicated that some APN(+) patients have been mis-subgrouped using the LNR system.

### Identification of the Optimal Cut-Off Values (0.15 and 0.34) for LNR-APN(−) Staging by X-Tile Analysis in APN(−) Patients With Positive Lymph Nodes Among 10,120 Breast Cancer Patients

**FIGURE 3:**
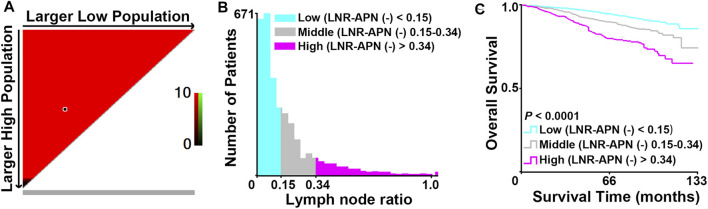
Identification of the optimal cut-off values (0.15 and 0.34) for LNR-APN(−) by X-tile analysis in APN(−) patients with positive lymph nodes among 10,120 breast cancer patients. **(A)** Red indicates a negative association. *X*-axis demonstrates all potential cut-off values from low to high (left to right), defined as larger low population. *Y*-axis demonstrates cut-off values from high to low (top to bottom), defined as larger high population. **(B)** Histogram of the entire cohort divided into three subgroups according to the optimal cut-off values of 0.15 and 0.34. **(C)** Kaplan–Meier curves showing the division of overall survival according to the cut-off values of 0.15 and 0.34 (*n* = 3,283, *p* < 0.0001).

In order to obtain the precise LNR, we focused on 3,283 APN(−) patients with positive lymph nodes from the 10,120 breast cancer patients and applied X-tile analysis to calculate two cut-off values (0.15 and 0.34) based on the OS of these patients (Figures 3A–C).

### LNR-APN(−) Staging Could Accurately Predict the Prognosis of APN(−) Breast Cancer Patients

**FIGURE 4:**
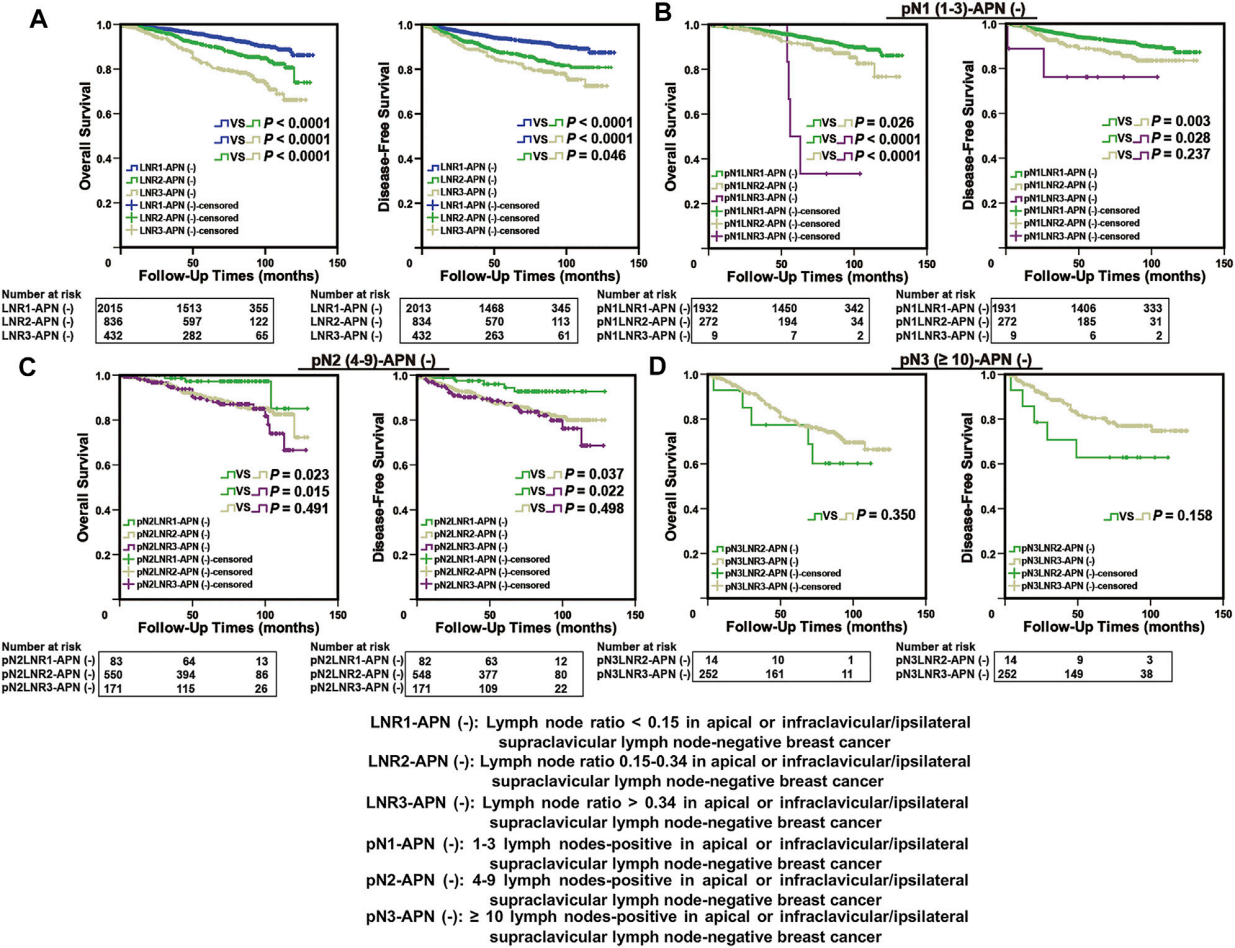
LNR-APN(−) could accurately predict the prognosis of APN(−) breast cancer patients. Kaplan–Meier analysis of **(A)** LNR-APN(−) breast cancer patients (*n* = 3,283), **(B)** pN1-LNR-APN(−) breast cancer patients (*n* = 2,213), **(C)** pN2-LNR-APN(−) breast cancer patients (*n* = 804), and **(D)** pN3-LNR-APN(−) breast cancer patients (*n* = 266).

The APN(−) breast cancer patients were classified into three groups based on the cut-off values and defined as LNR1-APN(−) (LNR > 0 and <0.15; *n* = 2015), LNR2-APN(−) (LNR ≥ 0.15 and ≤0.34; *n* = 836), and LNR3-APN(−) (LNR > 0.34; *n* = 432), which represented 52.1%, 24.0%, and 23.9% of patients in this study cohort, respectively. The groups categorized by LNR-APN(−) yielded a significant difference between the OS and DFS curves (*p* < 0.0001, Figure 4A). Consequently, LNR-APN(−) staging could predict the prognosis of breast cancer patients accurately.

Next, we divided pN1 breast cancer patients (*n* = 2,213) into three groups, namely, pN1-LNR1-APN(−), pN1-LNR2-APN(−), and pN1-LNR3-APN(−); pN2 breast cancer patients (*n* = 804) into pN2-LNR1-APN(−), pN2-LNR2-APN(−), and pN2-LNR3-APN(−); and pN3 breast cancer patients (*n* = 266) into pN3-LNR2-APN(−) and pN3-LNR3-APN(−). Survival analysis between different subgroups revealed that the pN1-LNR2-APN(−) and pN1-LNR3-APN(−) groups had a significantly worse prognosis than pN1-LNR1-APN(−) (*p* < 0.05, Figure 4B), and the pN2-LNR1-APN(−) group had a significantly better prognosis than the pN2-LNR2-APN(−) and pN2-LNR3-APN(−) groups (*p* < 0.05, Figure 4C). Moreover, pN1-LNR2-APN(−) and pN1-LNR3-APN(−) patients had a significantly worse prognosis than patients with pN1 stage, and pN2-LNR1-APN(−) patients had a better prognosis than patients with pN2 stage; however, there was no significant difference between LNR3-APN(−) and pN3 groups (Figure 4D; Supplementary Figure S1).

Multivariate analysis revealed LNR-APN(−) to be a better prognostic predictor of OS than pN-APN(−) in breast cancer by using the Cox proportional hazard regression model (*p* < 0.05). LNR2,3-APN(−) (LNR2-APN(−) and LNR3-APN(−)) breast cancer patients had a significantly worse OS than LNR1-APN (−) patients (HR = 1.843, *p* < 0.0001, Table 2).

**TABLE 2:** Overall survival multivariable analysis of APN(−) patients among 10,120 breast cancer patients.

Variable	HR	95% CI	*p* value
Age (years)			
<50	1	Reference	
≥50	1.378	1.085–1.749	0.009^**^
Histological grade			
I	1	Reference	
II	1.159	0.630–2.131	0.635
III	0.958	0.493–1.859	0.898
Estrogen receptor^a^			
Negative	1	Reference	
Positive	0.764	0.561–1.040	0.087
Progesterone receptor^a^			
Negative	1	Reference	
Positive	0.808	0.601–1.086	0.158
HER2 expression^a^			
0 and 1+	1	Reference	
2+	1.467	1.119–1.922	0.006^**^
3+	1.176	0.789–1.753	0.426
pT stage			
pT1	1	Reference	
pT2	1.699	1.269–2.276	<0.0001^***^
pT3	2.406	1.564–3.702	<0.0001^***^
pT4	6.413	3.677–11.185	<0.0001^***^
pN-APN(−)			
pN1-APN(−)	1	Reference	
pN2, 3-APN(−)	1.040	0.742–1.458	0.818
LNR-APN(−)			
LNR1-APN(−)	1	Reference	
LNR2, 3-APN(−)	2.006	1.424–2.826	<0.0001***

pN2, 3-APN(−): pN2-APN(−) and pN3-APN(−).

LNR2, 3-APN(−): LNR2-APN(−) and LNR3-APN(−).

**p < 0.01.

***p < 0.001, Cox regression analysis.

a Some data missing.

### Verify the Accuracy of the LNR-APN(−) System Using the SEER Database

**FIGURE 5:**
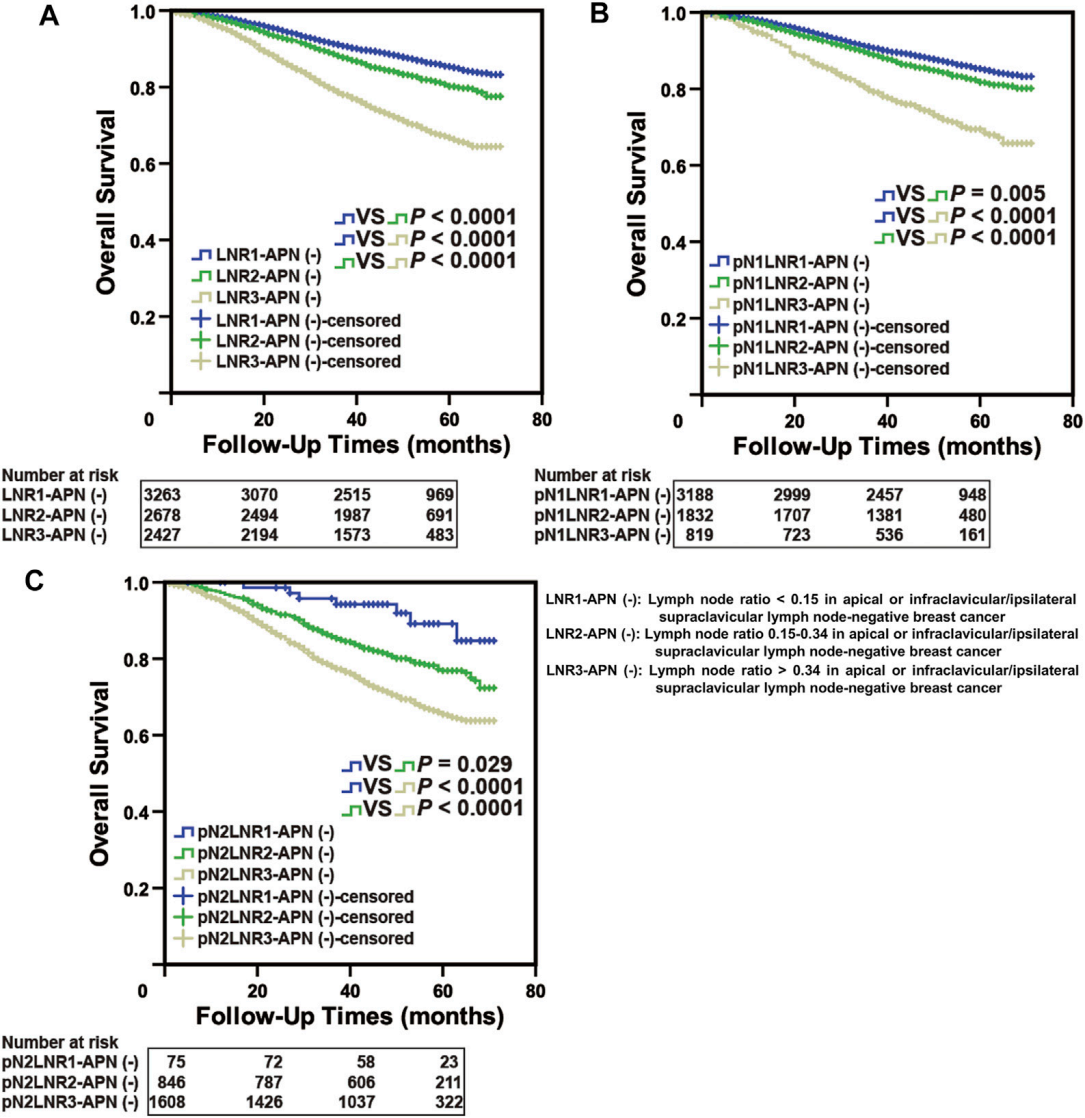
LNR-APN(−) could accurately predict the prognosis of pN1 and pN2 stage breast cancer patients in the SEER database. Kaplan–Meier analysis of **(A)** LNR-APN(−) breast cancer patients (*n* = 8,380), **(B)** pN1-LNR-APN(−) breast cancer patients (*n* = 5,846), and **(C)** pN2-LNR-APN(−) breast cancer patients (*n* = 2,534).

To further verify the accuracy of the LNR-APN(−) system in different clinical databases, we fixed our attention on the SEER database, which comprised 10,163 breast cancer patients. The clinicopathologic characteristics of the breast cancer patients are summarized in Supplementary Table S2. As information on the pathological features of the lymph nodes was unavailable in the SEER database, pN3 patients were excluded from further analysis. As expected, the groups categorized by LNR-APN(−) yielded a significant difference between the OS curves (*p* < 0.0001, Figure 5A). Moreover, pN1-LNR2-APN(−) and pN1-LNR3-APN(−) patients had a significantly worse prognosis than pN1-LNR1-APN(−) patients (*p* < 0.05, Figure 5B); pN2-LNR1-APN(−) and pN2-LNR2-APN(−) patients had a better prognosis than pN2-LNR3-APN(−) patients (*p* < 0.05, Figure 5C). The aforementioned results indicate that LNR-APN(−) could predict the prognosis of patients included in the SEER database.

### Neither the Published Cut-Off Values (0.2 and 0.65) nor Our Cut-Off Values (0.15 and 0.34) Could Accurately Predict the Prognosis of APN(+) Patients

**FIGURE 6:**
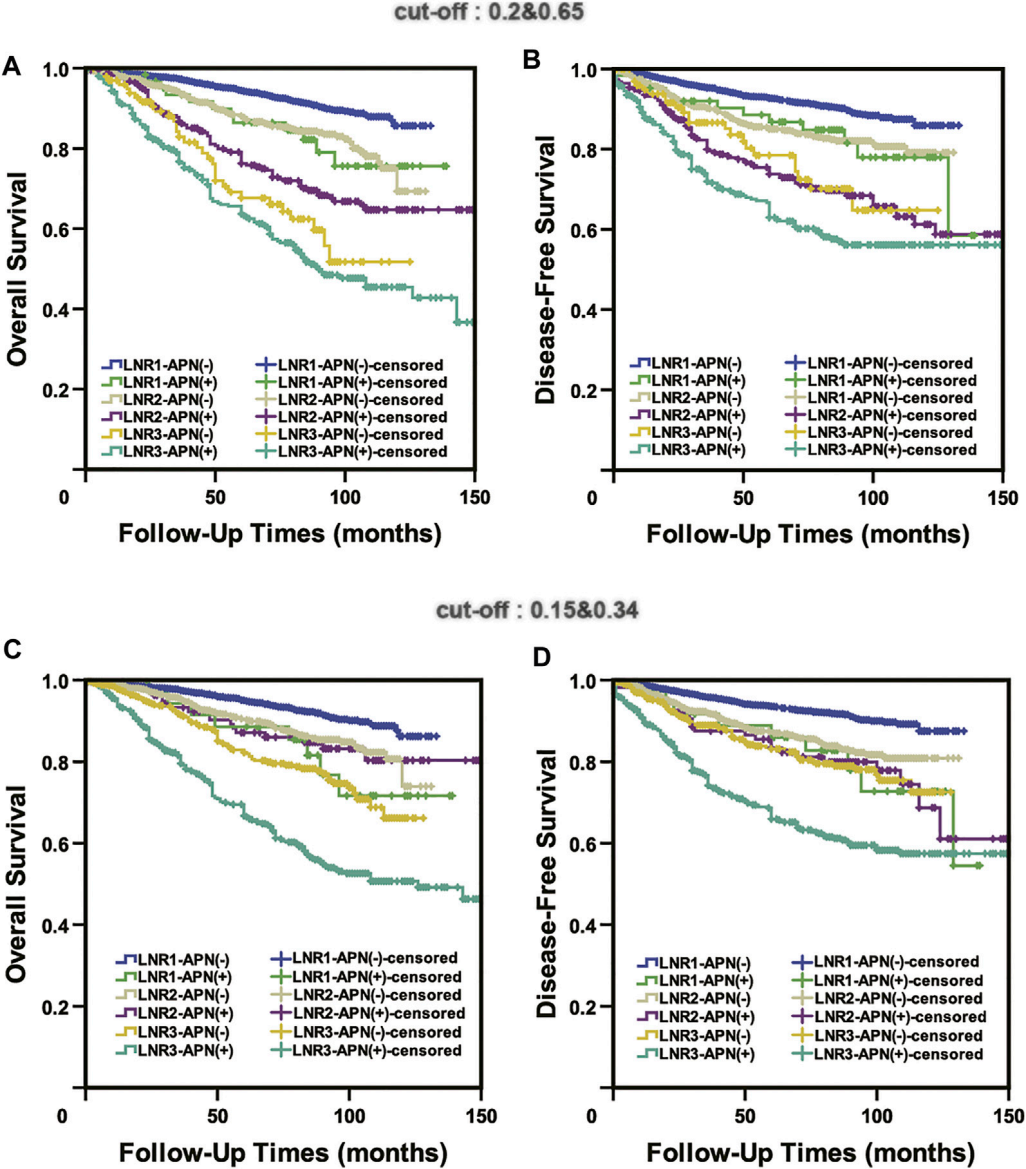
Neither the published cut-off values (0.2 and 0.65) **(A,B)** nor our cut-off values (0.15 and 0.34) **(C,D)** could accurately predict the prognosis of APN(+) patients.

We applied both the published cut-off values (0.2 and 0.65) and our cut-off values (0.15 and 0.34) to APN(+) patients, and the results indicated that none of them could accurately predict the prognosis of APN(+) patients. In the previously published system (0.2 and 0.65), there was no statistical difference in OS or DFS between LNR1-APN(+) and LNR2-APN(−) patients (OS: *p* = 0.842, DFS: *p* = 0.921) and between LNR2-APN(+) and LNR3-APN(−) patients (OS: *p* = 0.085, DFS: *p* = 0.636) (Figures 6A,B). There was also no difference in OS or DFS between LNR1-APN(+) and LNR2-APN(−) (OS: *p* = 0.402, DFS: *p* = 0.351) or LNR2-APN(+) patients (OS: *p* = 0.484, DFS: *p* = 0.955) in our system (0.15 and 0.34) (Figures 6C,D).

## Discussion

The current AJCC-TNM staging system classifies the pN stage based on only the number of positive lymph nodes. Over the past decades, increasing studies have suggested that the LNR system could be an accurate prognostic indicator in breast cancer, and LNR could be considered as an alternative to pN staging (Ahn et al., 2011; Ataseven et al., 2015; Chen et al., 2015; Solak et al., 2015; Cho et al., 2018). However, the AJCC-TNM staging system classified APN(+) breast cancer patients with a worse prognosis into the pN3 stage regardless of the lower-level lymph node metastasis state (Greene, 2002; Singletary et al., 2002). This point indicated a possibility that pN3-APN(+) patients with a small number of positive lymph nodes could be misclassified as low LNR stage. Until now, reports on LNR in breast cancer from different research groups have not mentioned this possibility using the LNR system (Dings et al., 2013; Yu et al., 2015; He et al., 2017; Ayşegül and Mehmet, 2020). Our results indicated that APN(+) patients had a significantly worse prognosis than APN(−) patients in the LNR1 (LNR ≤ 0.2) and LNR2 (LNR 0.21–0.65) groups, which strongly suggests that APN(+) patients should be excluded in the LNR system. In our study, we focused on 3,283 APN(−) patients with positive lymph nodes from among 10,120 breast cancer patients and applied X-tile analysis to calculate two cut-off values (0.15 and 0.34) based on the OS of these patients. Using these cut-off values, we classified our patients into LNR1-APN(−) (LNR > 0 and <0.15), LNR2-APN(−) (LNR ≥ 0.15 and ≤0.34), and LNR3-APN(−) (LNR > 0.34). We found that the LNR-APN(−) system could distinguish pN1-LNR2-APN(−) and pN1-LNR3-APN(−) patients with a significantly worse prognosis from pN1-LNR1-APN(−) patients to avoid inadequate treatment and could also distinguish pN2-LNR1-APN(−) patients with a significantly better prognosis from pN2-LNR2-APN(−) patients to avoid overtreatment, but had no role in identifying pN3 and LNR3-APN(−) patients. The study by Yu et al. (2015) suggested that LNR could be a significant prognostic factor in pN3 breast cancer patients. However, the study did not consider pN3 patients with or without APN(+) and did not compare the prognosis of subgroups of pN3 patients categorized by LNR with that of pN1 and pN2 patients. Therefore, the authors could not find the difference in the prognosis of subgroups of pN3 patients distinguished by LNR from that of pN1 and pN2 patients.

In our study, we applied the LNR system to APN(+) patients and compared their prognosis with that of other patients, and the results indicated neither the published cut-off values (0.2 and 0.65) nor our cut-off values (0.15 and 0.34) can accurately predict the prognosis of APN(+) patients. Despite the ethnic heterogeneity, the prognostic effect of LNR-APN (−) was successfully validated in another independent cohort from the SEER database. Due to the unavailability of data on the pathological features of the lymph nodes in the SEER database, pN3 patients were excluded from further analysis in this study. These results indicated that the LNR-APN(−) system could predict the prognosis of APN(−) patients accurately, and it may be a more comprehensive and valuable supplement to the previously reported LNR (Oven Ustaalioglu et al., 2010; Vinh-Hung et al., 2010; Xiao et al., 2013; Tonellotto et al., 2019). In the future, a comprehensive consideration of LNR and N staging may be a better choice when clinicians evaluate lymph node status in breast cancer patients.

Our cohort size of 10,120 breast cancer patients including 3,936 patients with positive lymph nodes, which comprise 3,283 APN(−) patients, is a large sample size, much larger than the sample size in comparable reports (Vinh-Hung et al., 2009; Danko et al., 2010; Li et al., 2012; Saxena et al., 2012; Liao et al., 2015), which makes our analysis more credible and representative. Moreover, uniform pathologic examination of the lymph node samples by a single institution ensures that similar surgical and pathologic procedures were performed. An additional advantage is a longer follow-up duration with a median of 81 months, which suggests that our data have a greater ability to predict the prognostic value of the variables being studied (Wang et al., 2012; Kim et al., 2016; Tsai et al., 2016; Wang et al., 2017). However, the retrospective nature of this study could have introduced bias in terms of patient and treatment selection. For this retrospective study to be meaningful, the baseline of patients such as sex, age, basic disease, and treatment cannot be considered. Individual differences exist objectively in any research and cannot be overcome one by one. It is an inherent disadvantage faced by any research. The way to minimize errors caused by treatment is to increase the sample size. Our study applied a breast cancer cohort of 10,120, which is a very large cohort size, even reaching the top of the international level. The cohort is large enough to ignore the errors caused by treatment. In addition, statistical analysis based on the Cox proportional hazards model showed that sample size has a significant impact on the results. To solve this problem, we should try more statistical methods or apply our cut-off values to another database for further validation.

## Conclusion

Our present study revealed that excluding APN(+) patients is the most necessary supplement to LNR and that LNR-APN(−) staging should be a more comprehensive approach in predicting prognosis and guiding clinicians to provide accurate and appropriate treatment.

## Data Availability

The raw data supporting the conclusion of this article will be made available by the authors, without undue reservation.

## Supplementary Material

Supplementary Figure S1 LNR-APN(−) could predict the prognosis of breast cancer patients more accurately than pN staging. Kaplan–Meier analysis of **(A)** LNR-APN(−) breast cancer patients (*n* = 3283), **(B)** pN1-LNR-APN(−) breast cancer patients (*n* = 2213), **(C)** pN2-LNR-APN(−) breast cancer patients (*n* = 804), and **(D)** pN3-LNR-APN(−) breast cancer patients (*n* = 266).
