# Supplementary material for: Breast Cancer Patients With Positive Apical or Infraclavicular/Ipsilateral Supraclavicular Lymph Nodes Should Be Excluded in the Application of the Lymph Node Ratio System
Source: Front Cell Dev Biol. 2022 Apr 4;10:784920. doi: 10.3389/fcell.2022.784920 (PMC9013846; doi:10.3389/fcell.2022.784920)
Supplement: Supplementary file 1 [file Table1.DOCX]

**Supplementary Table S1 Table Detailed description of abbreviation.**

| Abbreviation | Explanation |
| --- | --- |
| pN | Pathologic lymph nodes classification according to the sixth edition of AJCC staging system |
| APN (+) | Apical or infraclavicular/ipsilateral supraclavicular lymph nodes-positive breast cancer |
| APN (-) | Apical or infraclavicular/ipsilateral supraclavicular lymph node-negative breast cancer |
| pN-APN (-) | pN-Apical or infraclavicular/ipsilateral supraclavicular lymph node-negative breast cancer |
| pN1-APN (-) | 1-3 lymph nodes-positive in apical or infraclavicular/ipsilateral supraclavicular lymph node-negative breast cancer |
| pN2-APN (-) | 4-9 lymph nodes positive in apical or infraclavicular/ipsilateral supraclavicular lymph node-negative breast cancer |
| pN3-APN (-) | ≥ 10 lymph nodes-positive in apical or infraclavicular/ipsilateral supraclavicular lymph nodes-positive breast cancer |
| LNR | Lymph node ratio (positive lymph nodes number/total lymph nodes number) |
| LNR-APN (+) | LNR-Apical or infraclavicular/ipsilateral supraclavicular lymph nodes-positive breast cancer |
| LNR-APN (-) | LNR-Apical or infraclavicular/ipsilateral supraclavicular lymph node-negative breast cancer |
| LNR1-APN (-) | Lymph node ratio < 0.15 in apical or infraclavicular/ipsilateral supraclavicular lymph node-negative breast cancer |
| LNR2-APN (-) | Lymph node ratio 0.15-0.34 in apical or infraclavicular/ipsilateral supraclavicular lymph node-negative breast cancer |
| LNR3-APN (-) | Lymph node ratio > 0.34 in apical or infraclavicular/ipsilateral supraclavicular lymph node-negative breast cancer |
