# Supplementary figures and images for: Breast Cancer Patients With Positive Apical or Infraclavicular/Ipsilateral Supraclavicular Lymph Nodes Should Be Excluded in the Application of the Lymph Node Ratio System
Source: Front Cell Dev Biol. 2022 Apr 4;10:784920. doi: 10.3389/fcell.2022.784920 (PMC9013846; doi:10.3389/fcell.2022.784920)

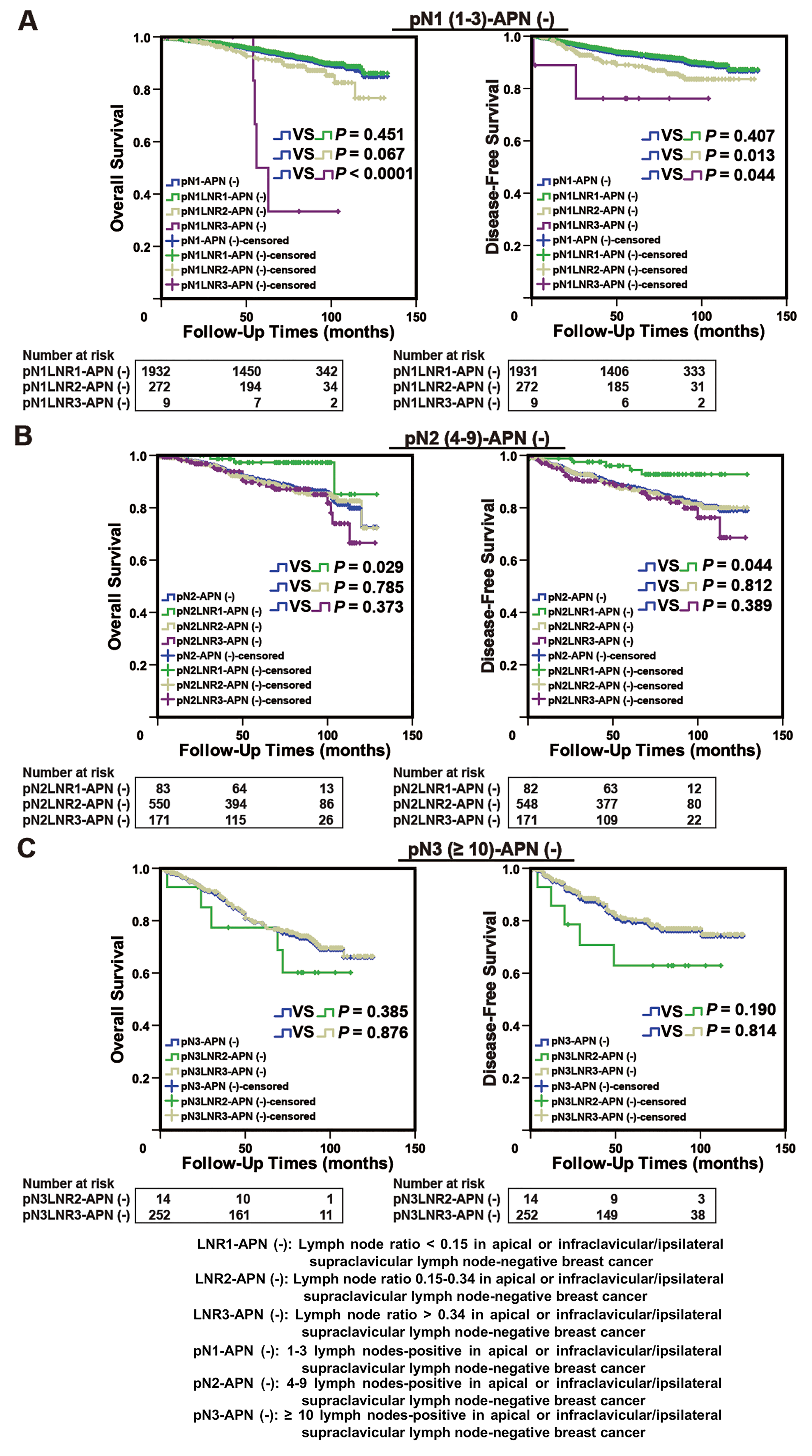

Supplement: Supplementary file 2 [file Image1.TIF]
