# Supplementary material for: Breast Cancer Patients With Positive Apical or Infraclavicular/Ipsilateral Supraclavicular Lymph Nodes Should Be Excluded in the Application of the Lymph Node Ratio System
Source: Front Cell Dev Biol. 2022 Apr 4;10:784920. doi: 10.3389/fcell.2022.784920 (PMC9013846; doi:10.3389/fcell.2022.784920)
Supplement: Supplementary file 3 [file Table2.DOCX]

**Supplementary Table S2 Table Clinicopathologic characteristics of breast cancer patients in SEER database (n = 10163).**

| Characteristic | Number of Patients  (n = 10163) | % |
| --- | --- | --- |
| Age |  |  |
| < 50 | 3150 | 31.0 |
| ≥ 50 | 7013 | 69.0 |
| Histopathologic type |  |  |
| Invasive ductal | 8472 | 83.4 |
| Invasive lobular | 1169 | 11.5 |
| Mucinous | 53 | 0.5 |
| Other types | 469 | 4.6 |
| Hormone receptor |  |  |
| Negative | 1969 | 19.4 |
| Positive | 8194 | 80.6 |
| HER2 gene |  |  |
| Negative | 8097 | 79.7 |
| Positive | 2066 | 20.3 |
| pT stage |  |  |
| pT1 | 2345 | 23.1 |
| pT2 | 4861 | 47.8 |
| pT3 | 1843 | 18.1 |
| pT4 | 1114 | 11.0 |
| Number of lymph nodes removed |  |  |
| 1-3 | 465 | 4.5 |
| 4-9 | 2029 | 20.0 |
| ≥ 10 | 7669 | 75.5 |
| pN stage |  |  |
| pN1 | 5846 | 57.5 |
| pN2 | 2534 | 24.9 |
| pN3 | 1783 | 17.5 |
